# Disruption of a cystine transporter downregulates expression of genes involved in sulfur regulation and cellular respiration

**DOI:** 10.1242/bio.017517

**Published:** 2016-05-03

**Authors:** Jessica A. Simpkins, Kirby E. Rickel, Marianna Madeo, Bethany A. Ahlers, Gabriel B. Carlisle, Heidi J. Nelson, Andrew L. Cardillo, Emily A. Weber, Peter F. Vitiello, David A. Pearce, Seasson P. Vitiello

**Affiliations:** 1 Biology Department, Augustana University, Sioux Falls, SD, USA 57197; 2 Sanford Research Children's Health Research Center, Sioux Falls, SD, USA 57104

**Keywords:** Cystine, *ERS1*, *CTNS*

## Abstract

Cystine and cysteine are important molecules for pathways such as redox signaling and regulation, and thus identifying cellular deficits upon deletion of the *Saccharomyces cerevisiae* cystine transporter Ers1p allows for a further understanding of cystine homeostasis. Previous complementation studies using the human ortholog suggest yeast Ers1p is a cystine transporter. Human *CTNS* encodes the protein Cystinosin, a cystine transporter that is embedded in the lysosomal membrane and facilitates the export of cystine from the lysosome. When *CTNS* is mutated, cystine transport is disrupted, leading to cystine accumulation, the diagnostic hallmark of the lysosomal storage disorder cystinosis. Here, we provide biochemical evidence for Ers1p-dependent cystine transport. However, the accumulation of intracellular cystine is not observed when the *ERS1* gene is deleted from *ers1*-Δ yeast, supporting the existence of modifier genes that provide a mechanism in *ers1*-Δ yeast that prevents or corrects cystine accumulation. Upon comparison of the transcriptomes of isogenic *ERS1+* and *ers1*-Δ strains of *S. cerevisiae* by DNA microarray followed by targeted qPCR, sixteen genes were identified as being differentially expressed between the two genotypes. Genes that encode proteins functioning in sulfur regulation, cellular respiration, and general transport were enriched in our screen, demonstrating pleiotropic effects of *ers1*-Δ. These results give insight into yeast cystine regulation and the multiple, seemingly distal, pathways that involve proper cystine recycling.

## Abstract (precis)

**Summary:** We identify genes that are differentially expressed in yeast lacking vacuolar cystine transporter Ers1p in order to find pathways, such as respiration and sulfur regulation, that are associated with cystine homeostasis.

## INTRODUCTION

Loss-of-function mutations in mammalian *CTNS* result in the absence of a cystine (oxidized dicysteine) effluxer, Cystinosin (Gahl et al., 2002; Kalatzis et al., 2001; Town et al., 1998). Without Cystinosin, the transport of cystine out of the lysosome is severely restricted, leading to cystine accumulation. Cystine is naturally found in the lysosome as result of protein hydrolysis and the influx of extracellular cystine (Danpure et al., 1986; Thoene and Lemons, 1980). Cystinosin exports cystine from the lysosome to the cytosol, where it can be reduced to cysteine to be used in downstream processes (Kalatzis et al., 2001). The absence of Cystinosin results in a surplus of cystine in the lysosome and eventual apoptosis (Jonas et al., 1982; Park et al., 2002; Schulman et al., 1969). Direct lysosomal dysfunction may contribute to cell death, but more likely, a lack of cystine recycling weakens the cell. For example, cysteine is the limiting precursor in glutathione synthesis, a tripeptide that functions in the elimination of oxidants that can damage DNA, proteins, and lipids. It is possible that apoptosis occurs secondarily to cystine storage, triggered by rampant reactive oxygen species that damage cellular components at higher rates due to a shortage of cysteine needed to synthesize sufficient levels of glutathione. In fact, lower levels of glutathione have been observed in cells lacking Cystinosin (Chol et al., 2004; Laube et al., 2006; Levtchenko et al., 2006; Mannucci et al., 2006). However, depleted ATP levels may also contribute to apoptosis (Coor et al., 1991; Kumar and Bachhawat, 2010; Levtchenko et al., 2006; Wilmer et al., 2008). There is also evidence that the lysosomes fragment and release cystine in mass into the cytosol, where the cystine is quickly reduced to cysteine. The large quantities of free cysteine then cysteinylate proapoptotic proteins, such as PKCδ (Park et al., 2002, 2006; Park and Thoene, 2005; Thoene, 2007). In addition, cystine accumulation may be affecting the cell in using a yet-uncharacterized mechanism. These mechanisms may not be mutually exclusive, and it is likely that a combination of these mechanisms is responsible for the observed increase in the rate of apoptosis in cells lacking Cystinosin.

The amino acid sequence of Cystinosin is 43% identical and 64% similar over 102 amino acids to a transmembrane protein encoded by *ERS1* in *Saccharomyces cerevisiae* (Town et al., 1998). The encoded yeast protein, Ers1p, localizes to the vacuole, an organelle analogous to the lysosome in mammalian cells (Gao et al., 2005). *ERS1* was originally identified as a high-copy suppressor of *erd1*-Δ, with *ERD1* encoding a protein necessary for ER protein retention, although the exact relationship between *ERS1* and *ERD1* remains unknown (Hardwick et al., 1990; Hardwick and Pelham, 1990). Deletion of *ERS1*, *ers1*-Δ, is lethal in the presence of the antibiotic hygromycin B in a strain-dependent manner. Human *CTNS* driven by the putative *ERS1* promoter complements *ers1*-Δ when the cells are grown in the presence of hygromycin B, indicating that proteins Ers1p and Cystinosin share common functions (Gao et al., 2005). The hygromycin B sensitivity can be suppressed by overexpression of *MEH1*, which encodes a protein involved in vacuolar acidification and general amino acid permease (Gap1p) localization (Gao and Kaiser, 2006; Gao et al., 2005).

Ers1p has been proposed to be a cystine transporter through complementation studies using *CTNS*, but Ers1p-dependent transport has not previously been reported (Gao et al., 2005). In this study, we performed a biochemical transport assay to confirm the existence of an Ers1p-dependent cystine transport system. Because human cells lacking Cystinosin accumulate 100-fold more cystine than normal cells do (Gahl et al., 2002; Schneider et al., 1967a,b), we measured cystine levels in *ers1*-Δ cells. Our results indicated, however, that *ers1*-Δ cells do not accumulate a significantly higher abundance of intracellular cystine compared to the *ERS1^+^* parental cells, and they show no difference in growth and survival. In this study we identified genes showing differential expression in *ers1*-Δ cells in order to understand pathways that are affected by a lack of vacuolar cystine transport in yeast.

## RESULTS

To better understand the primordial mechanisms of cystine regulation, *ERS1*, the gene encoding a putative cystine transporter, was deleted by homologous recombination, denoted *ers1*-Δ (Table S1). In all experiments, the BY4142 strains were used, including the control *ERS1^+^* parental strain, thereby eliminating changes that could be due to differences in auxotrophic markers.

### Ers1p-dependent cystine transport

**Fig. 1.**
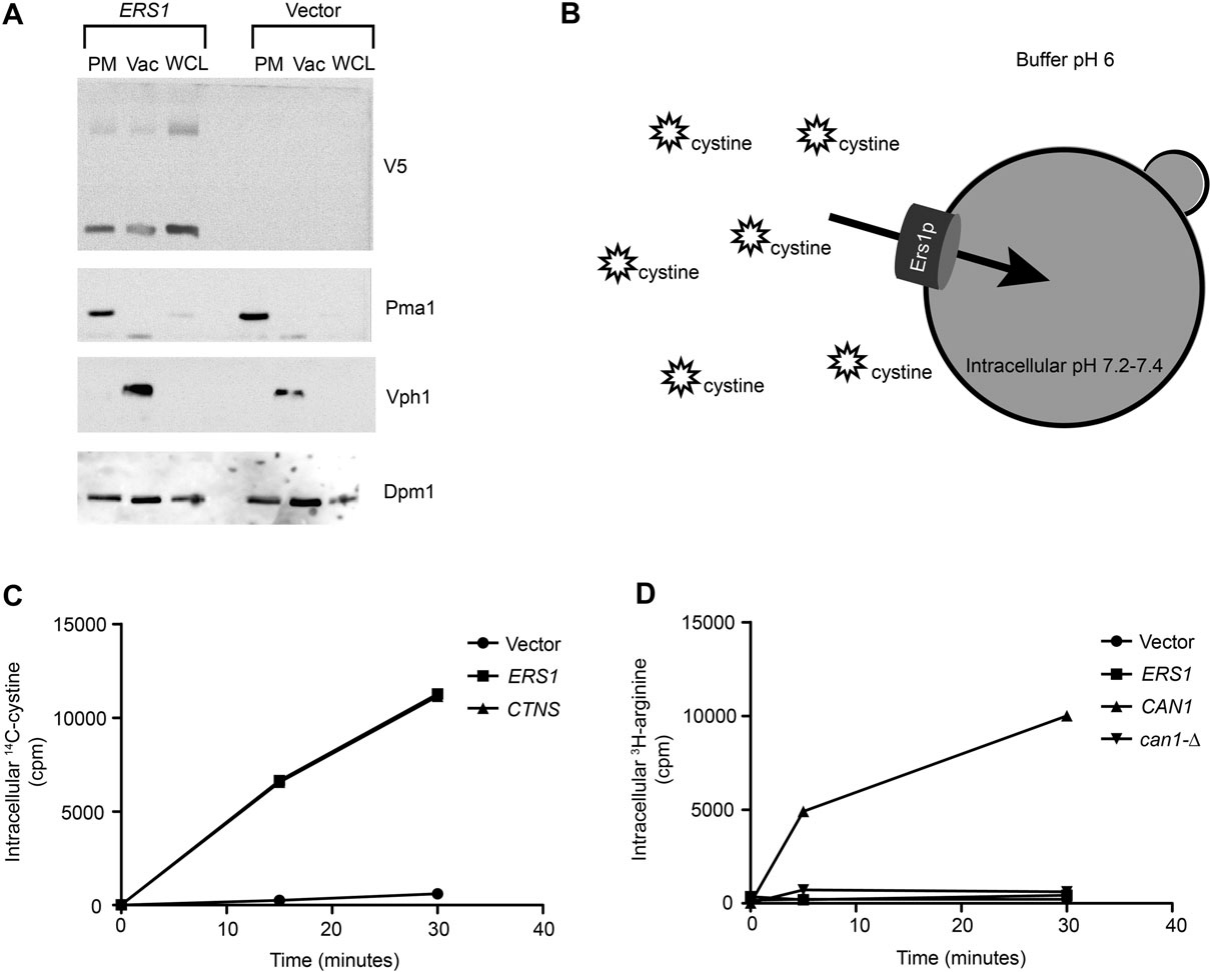
**Ers1p-dependent cystine transport.** (A) Western blot, representative of two replicates, showing that upon overexpression, Ers1p-V5 localizes to both the vacuole (Vac) and plasma membrane (PM). *ERS1*-*V5* was overexpressed using a galactose inducible promoter. Plasma membrane and vacuoles were enriched from whole cell lysate (WCL). Western blotting using an anti-V5 antibody indicated that Ers1p-V5 was located both in vacuoles (as determined by vacuolar marker Vph1p) and plasma membrane (as determined by plasma membrane marker Pma1p). The expected size of Ers1p-V5 was 30 kDa, with a larger band located at approximately 50 kDa. Dpm1p is an ER marker as an indicator of ER contamination. (B) Transport was measured by resuspending cells overexpressing either *ERS1* or *CTNS* in buffer at pH 6 and measuring the intracellular cystine over time. (C) Graph of Ers1p-dependent transport of cystine. Cells overexpressing *ERS1* or *CTNS* showed increased intracellular cystine over time, while cells with vector alone did not (mean±s.e.m.; *n*=4 cultures). (D) *ERS1* expression in *can1*-Δ did not rescue arginine transport, whereas *CAN1* expression did (mean±s.e.m.; *n*=4 cultures).

While previous studies have supported that *ERS1* and *CTNS* are orthologous, it had not been biochemically demonstrated that Ers1p transports cystine. We confirmed Ers1p-dependent cystine transport by creating an ‘inside-out’ vacuole model, in much the same way that Cystinosin-dependent cystine transport was previously measured (Kalatzis et al., 2001). When plasmid-derived *ERS1* is overexpressed in *ers1*-Δ cells (Table S2), Ers1p-V5 localizes to both the vacuole and plasma membrane with much of the protein detected in the fraction enriched for plasma membrane (Fig. 1A). Assuming that Ers1p is trafficked using standard protein transport mechanisms and that Ers1p transports cystine out of the vacuole, Ers1p bound in the plasma membrane would transport cystine into the cell. With this assumption, we resuspended the cells in an acidic buffer to mimic the pH of the vacuole, thereby establishing a proton-motive force required for transport (Fig. 1B). Over time, we observed an increase in intracellular radioactive cystine substrate in *ers1*-Δ cells overexpressing either *CTNS* or *ERS1* but not with vector alone, confirming that Ers1p can transport cystine *in vitro* (Fig. 1C). To confirm the specificity of Ers1p, we repeated the transport experiment using arginine, another amino acid commonly found in the vacuole. Arginine was transported into the yeast cells overexpressing *CAN1*, which encodes a plasma membrane arginine transporter (Grenson et al., 1966), but not *ERS1* (Fig. 1D). Previous work has also shown that endogenously expressed Ers1p localizes to the vacuole (Gao et al., 2005). All of these observations together support Ers1p-dependent cystine transport.

### Deletion of *ERS1* does not cause cystine accumulation or premature cell death

Human cells lacking Cystinosin accumulate approximately 100-fold more cystine than healthy controls (Schneider et al., 1967a,b). We measured cystine levels in *ers1*-Δ cells using a similar liquid chromatography-mass spectrometry/mass spectrometry (LC-MS/MS) protocol that is used to measure cystine concentration in human cystinotic cells (courtesy of Bruce Barshop, University of California at San Diego) (Chabli et al., 2007), but found that there is no statistical difference in cystine levels between *ERS1^+^* and *ers1*-Δ strains in various medias and growth phases (data not shown). Without reducing lysosomal cystine, human cells with loss-of-function *CTNS* mutations undergo apoptosis (Park et al., 2002, 2006). In line with the observations of others, we have observed that the growth and viability of *ers1*-Δ cells are equivalent to *ERS1^+^* cells, indicating that *ERS1* is not an essential gene under typical growth conditions (Gao et al., 2005); therefore, there could be a transcriptional response for *ers1*-Δ in normal growth conditions*.* Identification of these genes would allow for a better understanding of the pathways that are perturbed in *ers1*-Δ and thus what could be maintaining proper cystine homeostasis in the absence of a cystine transporter.

### Sixteen genes are differentially expressed in *ers1*-*Δ*

Previous synthetic lethal screens yielded numerous putative interactors (Costanzo et al., 2010). To identify which genes are differentially expressed and compensating for *ers1*-Δ*,* we compared the transcriptomes of BY4142-parental *ERS1^+^* and BY4142-derived *ers1*-Δ using a DNA microarray*.* As expected, *ERS1* expression was undetectable in the mutant. Laboratory yeast strains have auxotrophic markers that can be used as selectable markers. We did not see differences in expression of any of the strain-specific auxotrophic markers, *MET15*, for example, in the microarray results, indicating that the differences observed were due to *ers1*-Δ alone. Upon performing probe-based qPCR on each of the twenty-four genes that were identified via microarray, we confirmed sixteen genes that are differentially expressed in *ers1*-Δ. We considered a relevant fold-change as greater than a two-fold difference from the *ERS1^+^* parental, with *P*<0.05. Fourteen of the sixteen validated genes showed decreased expression in *ers1*-Δ, with only two genes having increased expression (Table 1). Fifteen of the sixteen gene expression changes were observed only in minimal media when the cells were grown to mid-log phase. However, one gene, *GEX1*, had decreased expression in *ers1*-Δ in rich media at mid-log phase and minimal media at either mid-log or stationary phase (Table 2), indicating a repression of *GEX1* expression in *ers1*-Δ, with *GEX1* encoding a glutathione exchanger (Dhaoui et al., 2011).

**Table 1.**
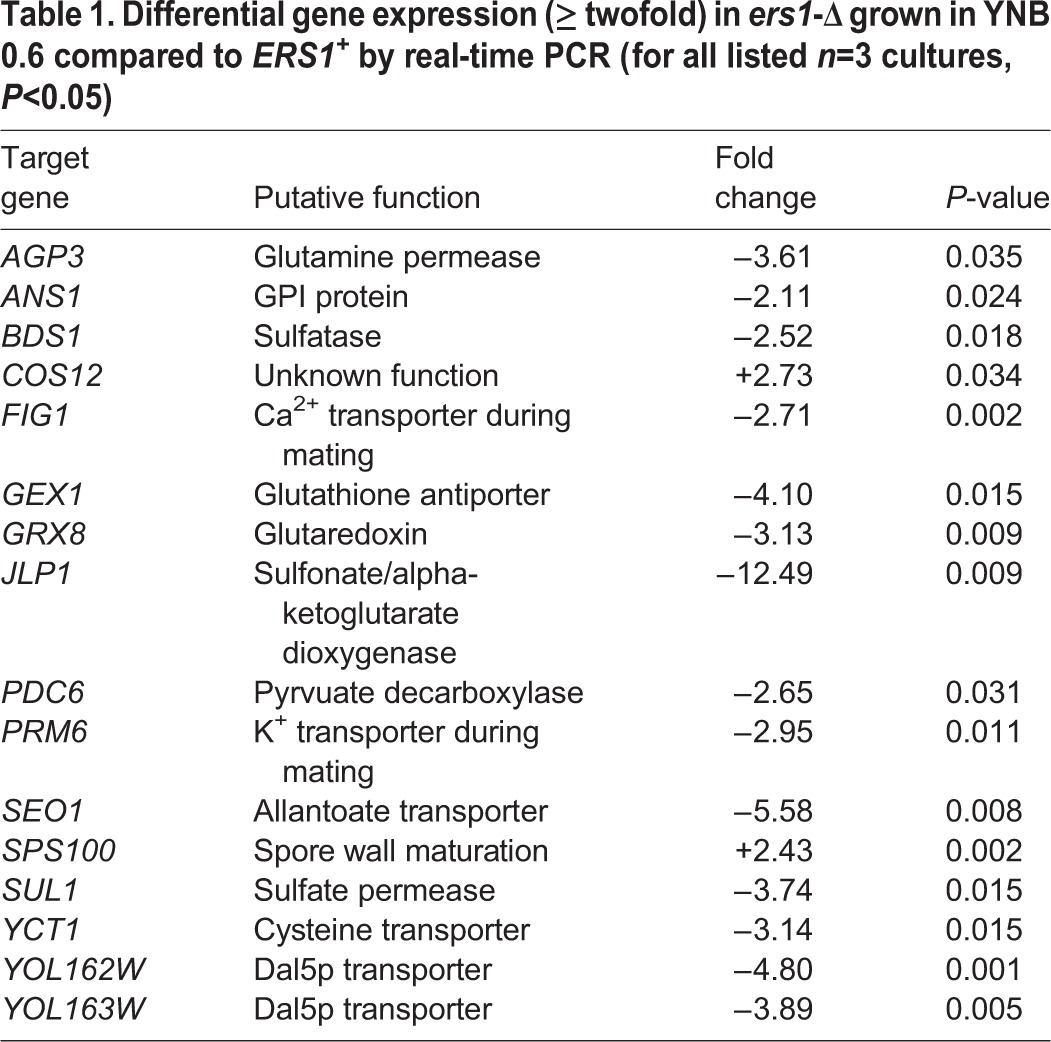
**Differential gene expression (≥ twofold) in *ers1*-Δ grown in YNB 0.6 compared to *ERS1^+^* by real-time PCR (for all listed *n*=3 cultures, *P*<0.05)**

**Table 2.**
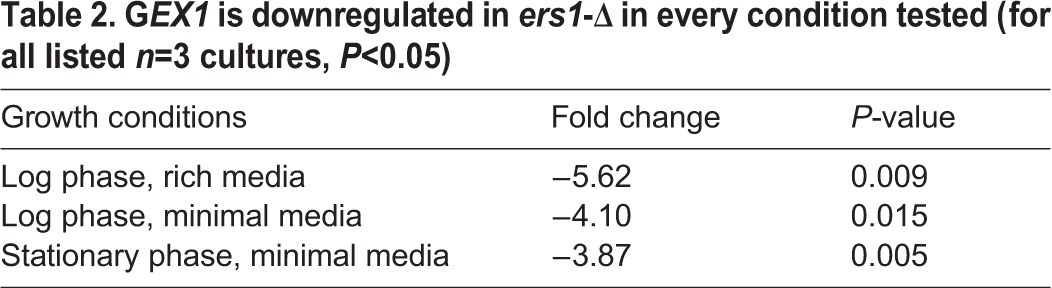
**G*EX1* is downregulated in *ers1*-Δ in every condition tested (for all listed *n*=3 cultures, *P*<0.05)**

**Fig. 2.**
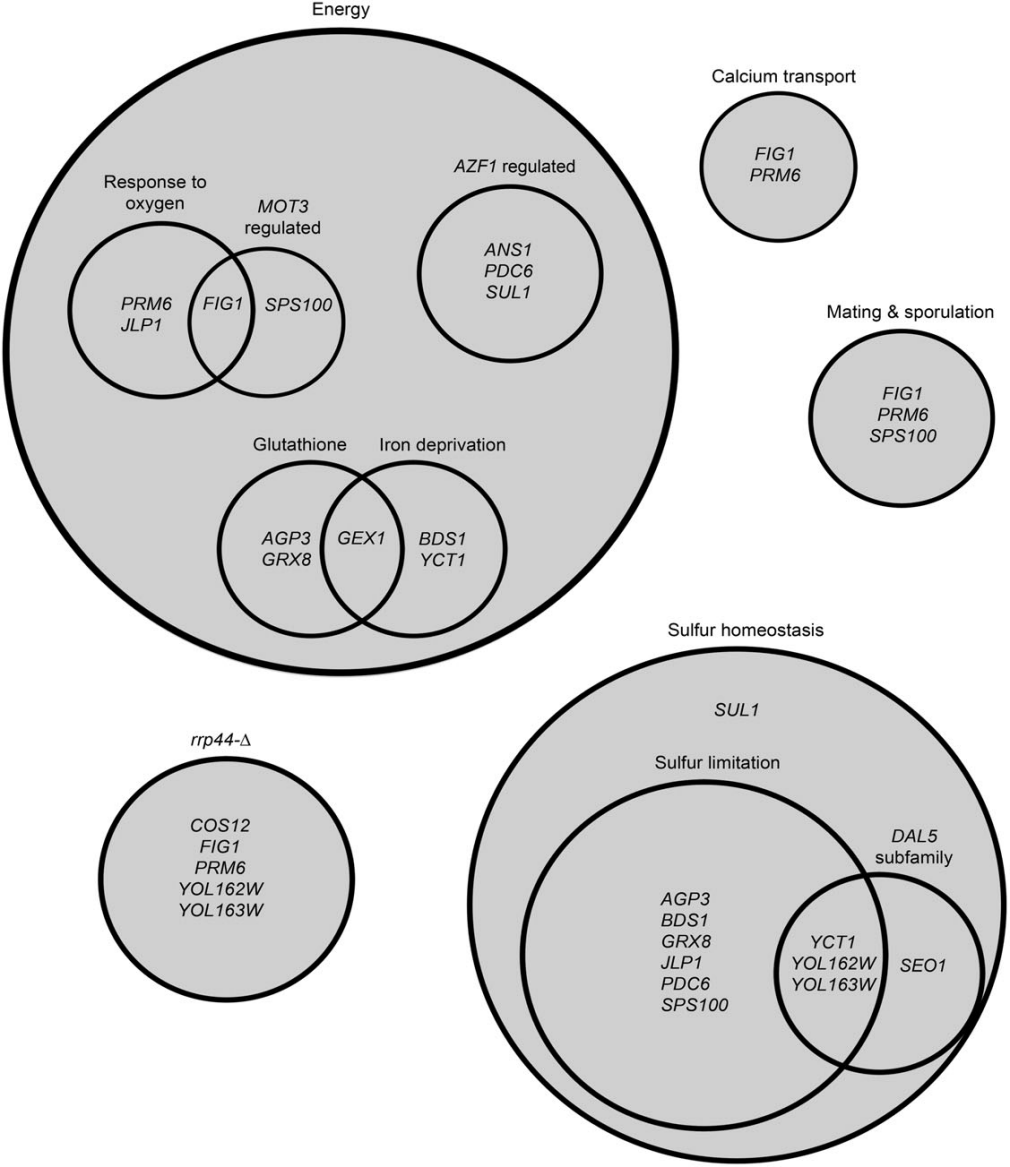
**Genetic interactions with *ERS1*.** Genes found to have differential expression in *ers1*-Δ were categorized into several functional groups, with energy and sulfur homeostasis as the two major groups. Most genes fell into more than one group.

The genes identified in the screen could be categorized into several key processes: cellular respiration and redox-related, sulfur regulation, and the *DAL5* subfamily of transporters (Fig. 2). Many of these genes are important in multiple overlapping cellular processes; for example, genes involved in sulfur regulation could also play a role in redox regulation. Genes involved in mating and sporulation and cell wall composition may be differentially expressed as a stress response, but can also be functionally categorized in the other groups.

### *ers1*-Δ exhibits increased aerobic respiration

**Fig. 3.**
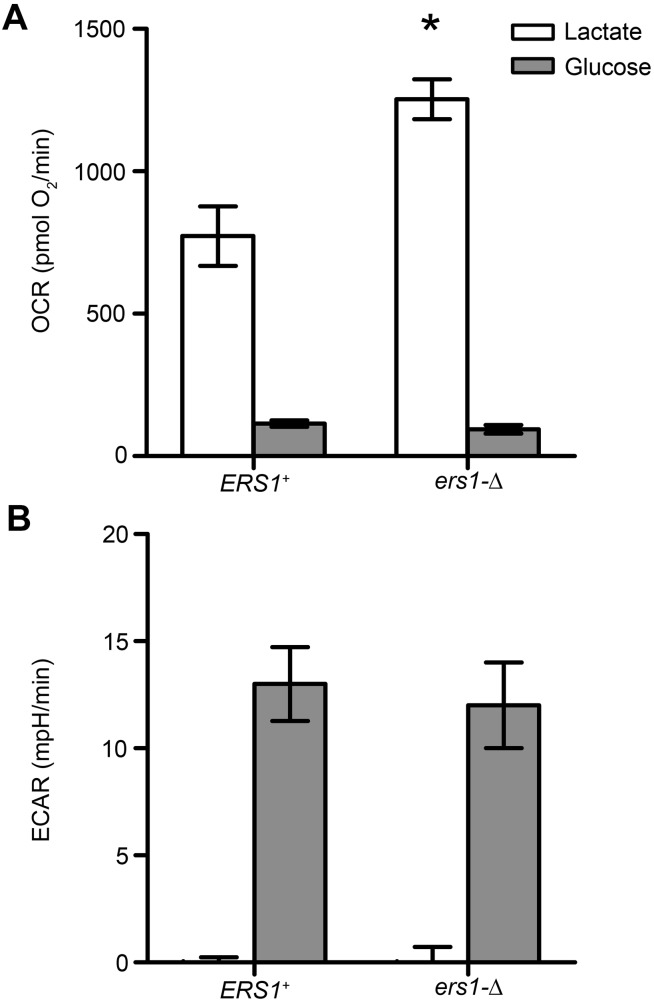
**Cells lacking *ERS1* have increased aerobic respiration *in vivo*.** Cells were grown to mid-log phase in minimal media and shifted to minimal media with either 2% glucose or 2% lactate. (A) Aerobic respiration is increased in media containing lactate, a non-fermentable carbon source, but not in glucose as per measurements using an FX Flux Analyzer. (B) Extracellular acidification was the same in the two genotypes in both carbon sources. Data represented as mean±s.e.m.; *n*=3 cultures. Statistical comparisons were made using Student's *t*-test (*P<0.05).

**Fig. 4.**
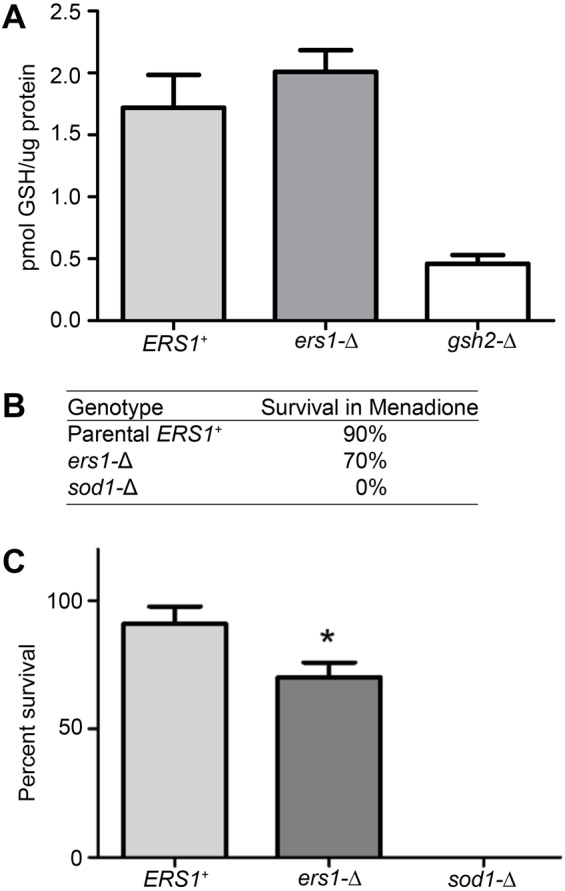
***ers1*-Δ cells have decreased survival in menadione and wild-type glutathione levels.** (A) The amount of glutathione (GSH) in cells grown to mid-log phase in minimal media was quantified (mean±s.e.m.; *n*=3 cultures). Since *GSH2* encodes the protein that catalyzing a major step in the glutathione pathway (Inoue et al., 1998), *gsh2*-Δ served as a control. (B) Cells (*n*=3 cultures) were incubated in 100 μM menadione or vehicle for 4 h, and then plated on rich media at 30°C. After 2 days, colonies were counted. Drug treatment was normalized to vehicle controls to calculate percent survival. Superoxide dismutase is encoded by *SOD1*. The deletion strain, which will not survive in menadione, was used as a control. (C) Graph of survival rates in B (mean±s.e.m.). Statistical comparisons were made using two-way ANOVA and Bonferroni post-test (*P<0.001).

Given that many genes related to cellular respiration were enriched in our screen, we measured aerobic respiration *in vivo* in our mutant. Using flux analysis, we observed that our mutant strain has increased respiration when grown in lactate, a non-fermentable carbon source; further suggesting that aerobic respiration is perturbed in *ers1*-Δ (Fig. 3). Although cellular respiration and redox function were the largest groups represented in our screen and we observed media- and growth phase-independent decreases in *GEX1* expression, total glutathione levels were normal in our mutant strain (Fig. 4A). However, we did observe a significant decrease in the ability of our mutant to survive in the oxidant menadione (Fig. 4B,C). Likewise, *ers1*-Δ sensitivity to hydrogen peroxide has been reported (Brown et al., 2006).

### Met28p and Azf1p binding sites are enriched in the promoter sequences of differentially expressed genes

While comparing the putative promoter sequences of the sixteen genes against sequences for common regulatory elements, we found that eleven of the genes contained a Met28p binding site in their promoter. A second motif was common in ten of the genes, identified as the binding site for Azf1p. All sixteen genes, with the exception of *ANS1*, had one or both motifs in their promoter sequence (Table 3). Met28p is a transcription factor that regulates expression of genes involved in sulfur regulation, while Azf1p is a transcription factor involved in carbon metabolism, further emphasizing the importance of these two functional categories in *ers1*-Δ (Kuras et al., 1996; Slattery et al., 2006).

**Table 3.**
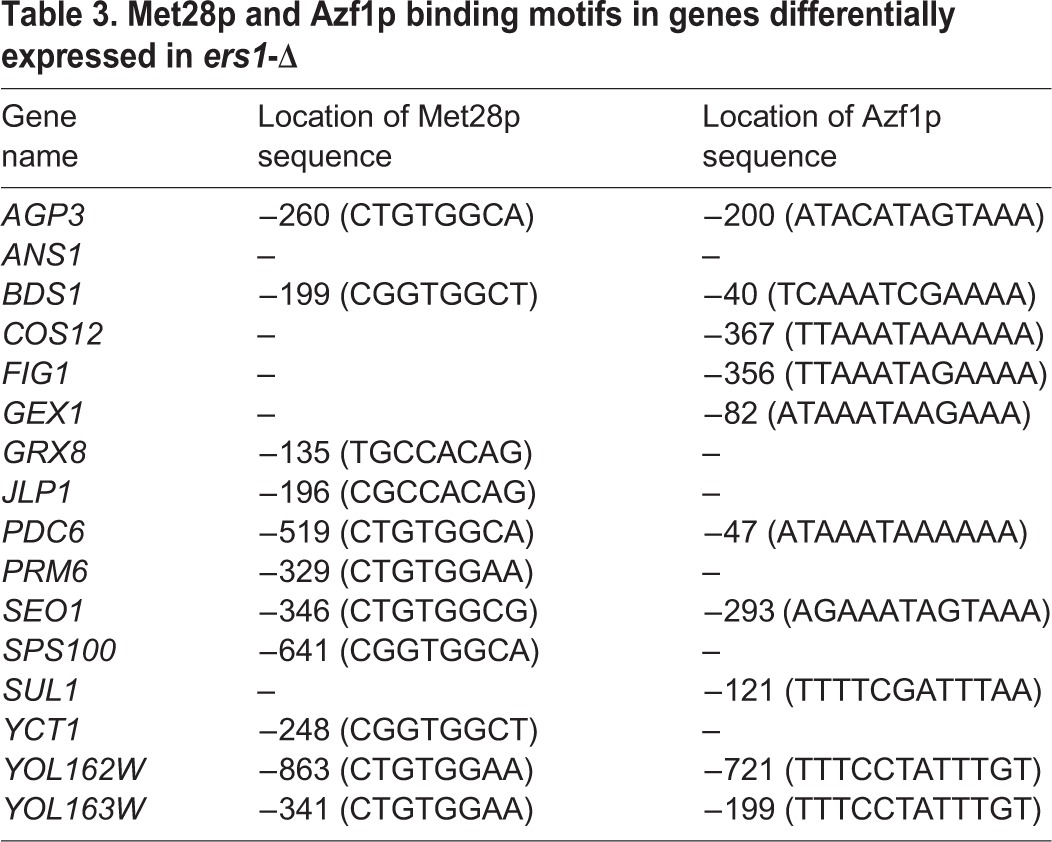
**Met28p and Azf1p binding motifs in genes differentially expressed in *ers1*-Δ**

## DISCUSSION

Comparison of the transcriptomes of *ERS1^+^* versus *ers1*-Δ yielded sixteen genes with differential expression. Two functional pathways, sulfur homeostasis and respiration, were over-represented in our data (Fig. 2). Similar pathways were observed when comparing the expression profiles of control and cystinotic cells from patients with nephropathic cystinosis (MIM 219800), an autosomal recessive lysosomal storage disorders resulting from loss-of-function *CTNS* mutations. They too uncovered genes involved in energy production and oxidative stress, as well as calcium and potassium transport, and stress response to hypoxia and glucose starvation. However, there were several represented pathways in cystinosis patient cells that were not observed in our model, such as immune function, cell-cycle regulation and apoptosis (Sansanwal et al., 2010). We present a set of sixteen genes involved in distinct functional pathways that have changed transcription in the mutant.

### Sulfur homeostasis

We found that eleven of the sixteen genes that are differentially expressed in *ers1*-Δ play a role in sulfur homeostasis. While some of these genes may not directly contribute to sulfur homeostasis, nine of the eleven genes were shown to have differential gene expression under sulfur limitation (Boer et al., 2003). Interestingly, promoter analysis showed that these nine genes changed under sulfur limitation also have a Met28p binding site. Met28p regulates sulfur metabolism, and is a part of the *MET4* complex, which is involved in general transcription regulation (Kuras et al., 1996). Although *ERS1* does not contain a putative Met28p binding site, cysteine metabolism is tightly linked with methionine synthesis. Eight genes (*AGP3*, *BDS1*, *GRX8*, *JLP1*, *PDC6*, *YCT1*, *YOL162W*, *YOL163W*) were upregulated under sulfur limitation but downregulated in our model. One other gene, *SPS100*, which was downregulated under sulfur limitation, was upregulated in our model. In summary, the nine genes previously found to be differentially expressed in sulfur limitation show the opposite expression profile in our model.

One of the eleven genes involved in sulfur homeostasis, *SUL1*, encodes a transporter that directs sulfate into the cytoplasm where it may be converted into homocysteine. Homocysteine is used in the synthesis of other thiols, such as cysteine and methionine (Jennings and Cui, 2012). *BDS1* closely relates to *SUL1* as it encodes a sulfatase that exploits a range of alkyl and aryl sulfates (Hall et al., 2005). Yct1p is a high-affinity cysteine transporter at the plasma membrane, and is key to maintaining cysteine homeostasis (Kaur and Bachhawat, 2007). Like *SUL1*, *SEO1* lacked differential expression under sulfur limitation. However, *SEO1* has a Met28p binding site in its promoter region, indicating that it plays a role in sulfur regulation. *SEO1*, along with *YCT1*, *YOL162W* and *YOL163W*, belongs to the *DAL5* subfamily of allantoate transporters (Llorente and Dujon, 2000). Expression of *SEO1* confers sulfoxide ethionine resistance, and it is thought that Seo1p may transport a sulfur compound that has yet to be identified (Isnard et al., 2002).The two uncharacterized open reading frames, *YOL162W* and *YOL163W*, have unknown function and unknown localization. According to our promoter analysis, *YOL162W* and *YOL163W* share a common Azf1p transcription factor binding site. Others have proposed that these two ORFs are one ORF, but perhaps the transcription of these two open reading frames, which are separated by only a few base pairs, share common transcriptional regulation since the a stop site between the ORFs is present according to the *Saccharomyces* genome database (Kellis et al., 2003). With the information that *YCT1* transports cysteine and the proposition that *SEO1* transports a currently unidentified sulfur compound, perhaps *YOL162W* and *YOL163W* also transport molecules important to sulfur metabolism and cystine regulation (Isnard et al., 2002; Kaur and Bachhawat, 2007). The last gene that fits concretely under the sulfur metabolism framework is *JLP1*, which is involved in sulfonate catabolism and is a sulfonate/alpha-ketoglutarate dioxygenase (Hogan et al., 1999).

The remaining genes that exhibit differential expression under sulfur limitation, with the exception of *SPS100*, are related to intermediates of cystine and sulfur homeostasis, such as glutamine and pyruvate. *AGP3* encodes a high-affinity glutamine permease, and it responds to changes in cellular and environmental conditions to maintain homeostasis of amino acids in the cell (Kaur and Bachhawat, 2007; Schreve and Garrett, 2004). *PDC6* is part of the pyruvate decarboxylase complex (PDC), which decarboxylates pyruvate to acetaldehyde in the cytoplasm and is the key enzyme involved in alcohol fermentation (Hohmann, 1991).

Cystine and cysteine are tightly linked to sulfate, sulfide, glutathione and pyruvate pathways. Observing that a sulfate transporter and a sulfatase are both downregulated in our model, we predict that extra sulfate and/or sulfur is produced in *ers1*-Δ, and these pathways are downregulated to correct for this excess.

### Energy and redox

Twelve of the sixteen genes that are differentially expressed in our model are related to energy metabolism and redox processes. Three genes showed altered expression in hypoxic environments: *PRM6*, *FIG1* and *JLP1*. *PRM6* and *FIG1* both show decreased expression in hypoxic and hyperoxic conditions, and both genes are involved in mating (Rintala et al., 2009). *PRM6* encodes a potassium transporter that promotes activation of the high-affinity calcium influx system (HACS). In yeast, HACS functions in promoting cell survival whereupon mating pheromones are present but potential mates are absent (Stefan et al., 2013). *FIG1* plays an important role in an additional calcium system known as the low-affinity calcium influx system (LACS). LACS promotes the fusion of cells during mating. Like that of *PRM6*, the expression of *FIG1* is induced through a signaling cascade instigated by pheromones (Muller et al., 2003). Interestingly, *FIG1* and another gene identified on our screen, *SPS100*, are both regulated by the transcription factor Mot3p, which is a repressor of hypoxic genes. Hypoxic genes are inhibited under aerobic conditions and activated upon oxygen limitation (Martinez-Montanes et al., 2013). *SPS100* is involved in spore wall maturation, and its protein may be a part of the glycoprotein matrix found in the spore wall (Law and Segall, 1988). The third gene affected by hypoxic conditions, *JLP1*, which encodes a sulfonate/alpha-ketoglutarate dioxygenase, shows sensitivity towards oxygen depletion (Hogan et al., 1999; Samanfar et al., 2013). Although the cells in our model were grown in normoxia, it is possible that *ERS1* plays a role in response to altered levels of oxygen in the cell. This may also be linked to the increased aerobic respiration that is observed in our model when cells are grown in lactate. Additionally, *JLP1* is inhibited by succinate, an intermediate of the citric acid cycle (Smith et al., 2007). Another gene identified on the screen, *PDC6*, is likely to contribute to pyruvate decarboxylase activity when grown in non-fermentable carbon sources, such as ethanol. PDC decarboxlyates pyruvate to acetaldehyde in the cytoplasm and is the key enzyme involved in alcohol fermentation (Hohmann, 1991).

Our promoter analysis indicated that ten genes on our screen (*AGP3*, *BDS1*, *COS12*, *FIG1*, *GEX1*, *PDC6*, *SEO1*, *SUL1*, *YOL162W*, *YOL163W*) contain binding sites for transcription factor Azf1p, which activates different sets of genes depending on which carbon source is present (Slattery et al., 2006). In our system, it is likely that Azf1p is activating genes involved in carbon and energy metabolism. *PDC6*, *SUL1* and *ANS1* all show decreased transcription when transcription factor *AZF1* is deleted and cells are grown in glycerol-lactate, a non-fermentable carbon source. *ANS1* transcription is also decreased in *azf1*-Δ when grown in glucose (Slattery et al., 2006). *ANS1* is not well characterized, and *in silico* analysis identified a putative GPI attachment signal within the amino acid sequence of Ans1p (Caro et al., 1997). When *ers1*-Δ cells were grown in lactate, we observed a difference in aerobic respiration compared to *ERS1^+^*; however when cells were allowed to ferment via growth on glucose, the difference in aerobic respiration was lost. This indicates that *ERS1* may play a role in energy metabolism in the cell, and that fermentation may better allow for *ers1*-Δ to circumvent dysfunction caused by a lack of Ers1p.

In mammalian cystinotic cells ATP depletion is observed (Coor et al., 1991; Wilmer et al., 2008); likewise, intracellular ATP concentration and respiration, as measured by oxygen consumption, is decreased in a model of cystinosis in which the cells are artificially loaded with cystine using CDME (Sakarcan et al., 1992). Yeast cells may be able to meet the energy demand as a consequence of fermentation and/or increased respiration. Additionally, redox regulation is tightly linked to aerobic respiration. Two of the genes identified in our screen, *GEX1* and *GRX8*, are directly involved in redox regulation. The former is a glutathione transporter, while the latter encodes a putative dithiol glutaredoxin (Dhaoui et al., 2011; Mesecke et al., 2008). First identified *in silico* as a hypothetical glutaredoxin-like protein, *GRX8* is the newest addition to the dithiol glutaredoxins in yeast (Fetrow et al., 2001; Mesecke et al., 2008). However, *GRX8* does not seem to be essential in the defense against oxidative stress. Additionally, although its structure contains a canonical Grx fold, the substrate binding site and catalytic center of Grx8 differ from other Grxs (Eckers et al., 2009). *GEX1* expression is induced under iron depletion (Dhaoui et al., 2011); likewise, *BDS1* and *YCT1* exhibit increased transcription under iron deficiency (Shakoury-Elizeh et al., 2010). The functionality of Jlp1p is also dependent on iron (Hogan et al., 1999).

Curiously, we noticed that five of the genes in our screen (*PRM6*, *FIG1*, *COS12*, *YOL163W*, *YOL162W*) showed differential expression when the exonuclease activity of the exosome was disrupted via a mutation in *RRP44* (Tsanova et al., 2014). More than sixty genes were identified as having greater than twofold increase in gene expression in *rrp44*-Δ; therefore a link to the exosome may simply be due to interactions with genes involved in redox regulation and respiration in our model.

Many of the genes uncovered in our screen are linked to multiple cellular pathways. Consequently, some of the pathways that are perturbed in our mutant may be due to pleiotropic effects. Uncovering the primary *ers1*-Δ suppressing pathway will be important for understanding the primordial mechanisms behind cystinosis, along with sulfur and respiration processes in yeast.

## MATERIALS AND METHODS

### Cell maintenance and growth

Unless otherwise described, cells were maintained, genetically manipulated, and grown using standard techniques and media conditions as previously described (Adams et al., 1997).

### Cell fractionation and western blotting

Mutant *ers1*-Δ cells (BY4742; GE Dharmacon-Open Biosystems, Huntsville, AL, USA) were transformed with pYes2.1 vector or pYes2.1 containing yeast *ERS1* or *CTNS* cDNA*.* These plasmids were kindly provided by Vincenza Dolce (University of Calabria, Arcavacata di Rende, CS, Italy) (Table S2). Cells were grown to stationary phase at 30°C in 2% glucose media, shifted to 2% galactose for 16 h, and harvested by centrifugation. Vacuoles were enriched using methods by Ohsumi and Anraku (1981). For plasma membrane enrichment using differential centrifugation, the cell wall was disrupted by bead beating in Tris chloride and EDTA buffer, pH 7.6 in the presence of 1 mM phenylmethylsulfonyl fluoride. Lysate in STE20 buffer (100 mM TrisCl pH 7.6, 50 mM EDTA, and 20% sucrose) was clarified from intact cells by centrifugation at 500× ***g*** for 5 min. Lysate was spun at 20,000× ***g*** for 30 min. The resulting pellet was resuspended in 0.5 ml STE20 and centrifuged using a gradient of (from bottom to top) 53.5%, 43.5%, and 20% sucrose in the above TE buffer at 100,000× ***g*** for 3 h. The layer between 53.5% and 43.5% sucrose was centrifuged in STE20 at 100,000× ***g*** for 1 h. The pellet was centrifuged again in the above sucrose gradient, followed by STE20. The final pellet containing plasma membrane was resuspended in 20 mM MES-Tris, pH 6.9 with 0.2 mM MgCl_2_. Total protein concentration of each fraction was measured by Bradford assay (Thermo Fisher Scientific-Pierce, Rockford, IL, USA). Protein (10 μg) was separated by SDS-PAGE and transferred to nitrocellulose for immunoblotting. Ers1p-V5 was detected using a V5 antibody (Thermo Fisher Scientific-Pierce; PIMA515253, 0.212 μg/ml). Plasma membrane and vacuole fraction purity was assessed using antibodies to Pma1p and Vph1p (Abcam, Cambridge, MA, USA; ab4645, 0.1 μg/ml and ab113683, 1 μg/ml), respectively. Antibody to Dpm1p, Dol-P-Man synthase of the endoplasmic reticulum membrane was used to confirm an even level of endoplasmic reticulum contamination across the samples (Abcam; ab113686, 4 μg/ml). Goat anti-mouse IgG(H+L), Human ads-HRP secondary antibody was used (Southern Biotech, Birmingham, AL, USA; 1031-05). Molecular weight determination was carried out using Precision Plus Protein™ WesternC™ Standards (Bio-Rad, Hercules, CA, USA). Blots were imaged using a UVP BioSpectrum 810 Imaging System and VisionWorksLS Image Acquisition and Analysis software (UVP, Upland, CA, USA).

### Cystine transport

Cystine transport was modelled as described previously in mammalian systems (Kalatzis et al., 2001). As above, mutant *ers1*-Δ cells with pYES2.1 vector, pYES2.1 with *CTNS*, or pYES2.1 with *ERS1* (kindly provided by Vincenza Dolce) were grown to *A*_600_ of 0.6, induced in 2% galactose, harvested, resuspended to 1.08×10^7^ cell/ml in PBS at pH 6.0, and 200 μl were pipetted onto a Whatman GF/C filter on a Millipore vacuum manifold for the 0 s/min time point. Radioactive ^14^C-cystine (Perkin-Elmer, Waltham, MA, USA) was added to 100 µM. To squelch the reaction, cells were collected at 0, 15, and 30 min using a vacuum manifold. Radioactivity on the filters was measured using a scintillation counter. Measurements were made over four replicates for each timepoint.

### Total cystine measurements

Cystine levels were measured courtesy of Bruce Barshop (University of California at San Diego), as previously described (Dohil et al., 2010). *ERS1^+^* and *ers1*-Δ cells were grown to log or stationary phase in minimal with 2% dextrose or rich media, harvested, and then lysed by freeze-thawing. Protein was precipitated by addition of sulfosalicylic acid and harvested by centrifugation. The protein pellet was solubilized in 0.1 N NaOH and the protein concentration measured by Bradford assay. Cystine in the supernatant was measured by tandem mass spectrometry (API 4000 LC/MS/MS; Applied Biosystems, Foster City, CA, USA) using stable-isotope dilution with deuterated d4-cystine as internal standard. Cystine and d4-cystine are measured using multiple reaction monitoring experiments for the transitions m/z 241.1→152.1 and m/z 245.1→156.1 respectively. Cystine levels were normalized to protein concentration and compared using Student's *t*-test.

### RNA isolation and cDNA synthesis

Bulk RNA from isogenic BY4742 *ERS1^+^* or *ers1*-Δ cells (GE Dharmacon-Open Biosystems) grown to mid-log phase (O.D. 0.6) in rich media (1% yeast extract, 2% peptone, and 2% dextrose), mid-log phase in minimal media (yeast nitrogen base with uracil, lysine, leucine, histidine and 2% dextrose), or stationary phase (O.D. 2.0) in minimal media was isolated using Life Technologies-Invitrogen RiboPure Yeast kit following the manufacturer's instructions. Following Turbo DNase treatment (Thermo Fisher Scientific-Ambion, Foster City, CA, USA) as per the manufacturer's instructions, quality of the RNA was analyzed using an Agilent 2100 electrophoresis bioanalyzer system (Agilent Technologies, Santa Clara, CA, USA). RNA (2 μg) was reverse transcribed to cDNA, including no reverse transcriptase controls, via the Life Technologies-Invitrogen High Capacity cDNA Reverse Transcription kit using random hexamer primers and following the manufacturer's protocol (Thermo Fisher Scientific-Life Technologies-Applied Biosystems, Carlsbad, CA, USA).

### DNA microarray

Measurements of transcript levels in yeast were performed in collaboration with the Sanford/Burnham Medical Research Institute Analytical Genomics Core (Lake Nona) using DNA microarray. Complementary DNA made from RNA (above) was labeled and hybridized to an Affymetrix gene chip (Yeast Genome 2.0 array, Affymetrix, Santa Clara, CA, USA). The analysis included three biological replicates, each with three technical replicates. Gene changes were deemed significant when there was greater than a twofold difference between the fluorescence intensities of the strains and FDR and *P*<0.05.

### qPCR

Comparative real-time RT-PCR via probe-based detection was performed using cDNA derived from *ERS1^+^* and *ers1*-Δ cells as described above. Primers were designed using Beacon Designer version 7.01 (Premier Biosoft). Each reaction consisted of transcript-specific primer pairs and a FAM-labeled primer probe (Eurofins MWG Operon, Huntsville, AL, USA;Table S3), template cDNA, and Thermo Scientific Absolute Blue qPCR master mix (Thermo Fisher Scientific, Waltham, MA, USA), as per the manufacturer's guidelines, with at least three biological replicates and three technical replicates per plate along with no reverse transcriptase and no template controls. Thermal cycling and fluorescence detection was performed via a Stratagene Mx3000p real-time PCR instrument (Agilent Technologies-Stratagene, La Jolla, CA, USA). Following normalization of Ct values to the *ACT1* housekeeping transcript, target transcript changes were deemed significant via Student's *t*-test if *P*<0.05 and the fold-change was greater than ±2.0.

### *In silico* analysis of promoters

To identify regulatory elements present within the promoter region (1 kb upstream) of the sixteen identified genes, a program called HOMER (hypergeometric optimization of motif enrichment) was used. Utilizing a set of background sequences from *Saccharomyces cerevisiae* in order to eliminate random chance, HOMER uses probability matrices to determine motifs that are overrepresented in the target sequences and nucleotide occurrence. All sixteen gene sequences were obtained from the *Saccharomyces* Genome Database and analyzed from 1 kb upstream of the +1 transcriptional start site. Results were confirmed using the database of genes at the *Saccharomyces* Genome Database.

### Cellular respiration

Following culture in minimal media supplemented with 2% glucose or 2% lactate, 1×10^6^ cells were loaded onto XF24 microplates coated with 20 μl poly-D-lysine (2 mg/ml) and centrifuged at 50 ***g*** for 1 min. Oxygen consumption rate (OCR) and extracellular acidification rate (ECAR) were measured using the XF24 Flux Analyzer (Seahorse Bioscience, North Billerica, MA, USA) with the following protocol at 30°C in replicates of six per assay: MIX 1 min; WAIT 2 min; MEASURE 2 min. Measurements were made over three separate experiments. Statistical comparisons between *ERS1^+^* and *ers1*-Δ were made using Student's *t*-test.

### Glutathione quantification

Yeast were grown to mid-log phase in minimal media with 2% glucose. Cells (1.2×10^9^) were harvested. Protoplasts were made by incubating cells with zymolyase 20 T in 1.2 M sorbitol, 20 mM potassium phosphate buffer, pH 7.6 for 90 min. Protoplasts were harvested, resuspended in 500 μl of 5% metaphosphoric acid and spun at 1200× ***g*** for 10 min at 4°C. Half of the supernatant with 0.1 N NaOH was tumbled overnight at 4°C. Protein concentration was measured using Pierce BCA Protein Assay Kit (Thermo Fisher Scientific). Total glutathione was measured in the remaining supernatant using a Glutathione Assay Kit (Cayman Chemical, Ann Arbor, MI, USA), following the manufacturer's guidelines. The assay included three biological replicates, each with three technical replicates. Statistical comparisons between *ERS1^+^*, *ers1*-Δ, *gsh2*-Δ biological replicates were made using one-way ANOVA.

### Cell survival in menadione

Yeast cells were grown to mid-log phase in rich media (YPD) and diluted to 3.06×10^6^ cells/ml in potassium phosphate buffer, pH 6.3 with 0.1% dextrose. An aliquot of the cells was diluted 10^−3^ in water and plated onto three YPD plates for the zero timepoint. To the remainder of the culture, menadione to 100 μM or vehicle (98% ethanol) was added. Cells were incubated at 30°C and plated as above after 120 and 240 min. Colonies were counted after two days of incubation at 30°C. The average of the three plates was normalized to the zero timepoint. The exposure was repeated two additional times (*n*=3). Statistical comparisons between *ERS1^+^* and *ers1*-Δ were made using two-way ANOVA and Bonferroni post-test (*P<0.001).
